# Editorial for the Special Issue “Satellite DNA Genomics”

**DOI:** 10.3390/genes15091223

**Published:** 2024-09-18

**Authors:** Manuel A. Garrido-Ramos, Miroslav Plohl, Eva Šatović-Vukšić

**Affiliations:** 1 Departamento de Genética, Facultad de Ciencias, Universidad de Granada, 18071 Granada, Spain; mgarrido@ugr.es; 2 Division of Molecular Biology, Ruđer Bošković Institute, Bijenička 54, 10000 Zagreb, Croatia; miroslav.plohl@irb.hr

A significant portion of eukaryotic genomes consists of non-coding repetitive DNA sequences arranged in tandem arrays, known as satellite DNA (satDNA) [1]. Long-term research in the field of satDNA biology and recent advancements in genomic and bioinformatics tools have greatly expanded our understanding of these sequences. Current research in satDNA genomics has enabled important insights into the origin, connection with transposable elements, genome distribution patterns, and evolution of satDNAs. Additionally, the roles of satDNAs in centromere organization, heterochromatin assembly, chromosome stability, and gene expression regulation are becoming increasingly elucidated [2]. This Special Issue, “Satellite DNA genomics”, encompasses different research and review papers furthering and/or summarizing our knowledge on satDNAs from a genomics perspective.

Several papers in this Special Issue are based on the use of omics (satellitomics and repeatomics) approaches to study the content and organization of repetitive sequences [3,4,5,6]. Gržan et al. [3] discovered 46 novel low-copy satDNAs in the genome of the red flour beetle *Tribolium castaneum*. The annotations on the current assembly revealed that many of the satDNAs were organized into short arrays, having transposable elements in their vicinity, and some of them also had numerous repeat units scattered throughout the genome.

Silva et al. [4] explored 23 genomes of *Drosophila* species from the *montium* group to reveal their satDNA landscape. They identified 101 non-homologous satDNA families in this group, 93 of which were described for the first time. It was shown that there is no significant correlation between the satDNA content and genome size across the 23 species. The authors found that at least one satDNA originated from an expansion of the central tandem repeats present inside a Helitron transposon.

Gutiérrez et al. [5] have characterized the satellitome of the Aquitanian mole *Talpa aquitania*, which presented 16 different families, including telomeric sequences. The satDNA analysis indicated that they have different grades of clusterization, homogenization, and degeneration. Most of the satDNA families were also present in the genomes of other *Talpa* species. In the genomes of more distant Talpidae species, only some satDNAs were present, in accordance with the library hypothesis. Chromosomal localization by FISH revealed that some satDNAs are localized preferentially in centromeric and non-centromeric heterochromatin in *T. aquitania* and sister species.

Amosova et al. [6] performed comparative analyses of repetitive sequences found in the genomes of the subpolar and polar grass species *Deschampsia sukatschewii*, *Deschampsia cespitosa*, and *Deschampsia antarctica*. In the studied species, class I transposable elements constituted the majority of the repetitive DNA, while 12–18 high-confidence and 7–9 low-confidence satDNAs were identified. Chromosomal mapping of the rDNA and satDNAs enabled the construction of species karyograms and the detection of new molecular chromosome markers important for *Deschampsia* species.

Subirana and Messeguer [7] studied, for the first time, the transcription of an abundant satDNA in the bacterium *Bacillus coagulans* and in the nematode *Caenorhabditis elegans*. The RNA-seq results revealed the transcription of satellites in both species, and that interspersed satDNAs are transcribed in different tissues.

Four review papers are also included in this Special Issue, encompassing various aspects of satDNAs.

Šatović-Vukšić and Plohl [8] explored the advances in sequencing methodologies and specialized bioinformatics tools that enable the definition of all satDNAs in a genome (satellitome). A comparison of the satellitomes’ properties across different animal and plant species shows significant diversity and a large range of complexity levels. Differences exist not only in the number and abundance of satDNAs but also in their distribution across the genome, array length, interspersion patterns, association with transposable elements, and localization in heterochromatin and/or in euchromatin. The authors also summarize the organizational forms and evolutionary processes that may lead to satellitomes’ diversity, and revisit some basic notions regarding repetitive DNA landscapes in eukaryotic genomes.

Logsdon and Eichler [9] review how our understanding of the genetic architecture and epigenetic properties of human centromeric DNA have advanced. They note that preliminary studies of human and nonhuman ape centromeres reveal complex, saltatory mutational changes organized around distinct evolutionary layers. The authors discuss how pockets of regional hypomethylation within higher-order α-satellite DNA define the site of kinetochore attachment in all human chromosomes, although such epigenetic features can vary even within the same chromosome. They remark that the improving sequence resolution of satDNA is providing new insights into centromeric function, with potential implications for improving our understanding of human biology and health.

The review by Jenner et al. [10] focuses on non-model organisms and accessible experimental and *in silico* methods for identifying telomere and satDNA repeats. They also provide advice on how to perform and analyze such experiments and include common pitfalls. Applying research techniques that have previously been used to study non-model organisms appears to be a promising source of new information. The authors use many examples from plants and insects, in addition to humans and yeast, which are traditional models in telomere research.

Focusing primarily on mammals, the review by Vourc’h et al. [11] delves into the non-coding genomic sites directly bound by Heat Shock Factor 1 (HSF1), and describes the molecular function of the long non-coding RNAs (lncRNAs) produced in response to HSF1 binding. The described non-coding genomic targets of HSF1 are various sequences, including pericentric satDNA repeats. The authors believe that studying the evolutionarily conserved heat stress response has the potential to emerge as a powerful cellular tool to study lncRNAs produced from repeated or unique DNA regions.

In conclusion, this Special Issue explores various aspects, including the evolutionary patterns and functional implications, of satDNA sequences, providing insights into genomic architecture, diversity, and complexity from the perspective of satDNA.
